# Active Packaging Films Made by Complex Coacervation of Tragacanth Gum and Gelatin Loaded with Curcumin; Characterization and Antioxidant Activity

**DOI:** 10.3390/foods11203168

**Published:** 2022-10-11

**Authors:** Fateme Amani, Atefe Rezaei, Hajar Akbari, Cristian Dima, Seid Mahdi Jafari

**Affiliations:** 1 Nutrition and Food Security Research Center, Department of Food Science and Technology, School of Nutrition and Food Science, Isfahan University of Medical Sciences, Isfahan P.O. Box 81746-73461, Iran; 2 Faculty of Food Science and Engineering, “Dunarea de Jos” University of Galati, “Domnească” Str. 111, Building F, Room 107, 800201 Galati, Romania; 3 Department of Food Materials and Process Design Engineering, Gorgan University of Agricultural Sciences and Natural Resources, Gorgan P.O. Box 49138-15739, Iran; 4 Nutrition and Bromatology Group, Department of Analytical Chemistry and Food Science, Faculty of Science, Universidade de Vigo, E-32004 Ourense, Spain; 5 College of Food Science and Technology, Hebei Agricultural University, Baoding 071001, China

**Keywords:** tragacanth gum, gelatin, curcumin, complex coacervates, biodegradable packaging

## Abstract

The development of biopolymer-based green packaging films has gained remarkable attention in recent years. In this study, curcumin active films were prepared using different proportions of gelatin (GE) and a soluble fraction of tragacanth gum (SFTG) (1GE:1SFTG and 2GE:1SFTG) by complex coacervation. The various ratios of used biopolymers did not significantly impact the mechanical properties, thickness, and WVP of final films. However, biopolymers’ ratio impacted the moisture content, water solubility, swelling ratio, and release rate. Blending curcumin with biopolymers caused a reduction in tensile strength (from 1.74 MPa to 0.62 MPa for film containing 1GE:1SFTG and from 1.77 MPa to 0.17 MPa for film containing 2GE:1SFTG) and proliferation in elongation at break (from 81.48% to 122.00% for film containing 1GE:1SFTG and from 98.87% to 109.58% MPa for film containing 2GE:1SFTG). Moisture content and water solubility of films experienced a decrease after the addition of curcumin. Antioxidant activity of curcumin-loaded films was almost five times higher than neat film samples. Furthermore, the interreaction between the carboxylic group of SFTG and amide I of GE formed an amide linkage and was proven by FTIR analysis. TGA showed a drop in the thermal stability of film samples compared to the main ingredients. In general, the complex coacervate of SFTG and GE has the advantage of developing eco-friendly and low-cost packaging film in the food industry, especially for the protection of fatty foods.

## 1. Introduction

The adverse effects of using synthetic polymers and plastics in food packaging has raised concerns about environmental contamination and endangering wildlife. In addition, toxic chemical constituents of plastic packaging may leach into food and cause devastating effects on human health. Biopolymer-based packaging has been considered as a potential eco-friendly and nontoxic alternative to plastic films [1,2,3]. Different bio-based materials (protein, polysaccharide, and lipids) have the potential to be developed into biodegradable packaging materials [4]. 

Gelatin (GE) is known as a safe, low-price, and biodegradable polymer with film-forming properties. Additionally, GE can easily absorb UV light because of aromatic amino acids in its structure [5]. Due to some drawbacks, such as low thermal stability and poor mechanical and barrier properties, this biopolymer should be combined with other natural ingredients to present more desirable qualities [6]. Tragacanth gum (TG) is an acid-resistant anionic biopolymer obtained from dried sap of *Astragalus* species. It contains water-swellable and water-soluble parts. The hydrophilic nature of TG causes stabilizing and emulsifying properties and makes it a suitable constituent in the food and drug industries [7,8,9]. 

Proteins and polysaccharides can interact noncovalently and form coacervates or soluble complexes. The main driving forces for the noncovalent bonds are electrostatic, van der Waals, hydrogen bonding, and hydrophobic interactions [10]. Since most natural polysaccharides have negative charge and proteins have a positive charge at the pH values lower than their isoelectric point, stronger complexations of proteins and polysaccharides are expected [11]. Coacervation is the separation into two liquid phases in colloidal systems. The more concentrated phase is the coacervate, and the equilibrium solution is the other phase [12]. According to previous studies, complex coacervation is a potential technique in food packaging [13,14,15]. 

Due to the increasing concern of consumers over various side effects of synthetic preservatives, current studies have focused on producing active packaging films loaded with natural bioactive compounds including essential oils, plant extracts, and curcumin. Curcumin is the main polyphenolic compound in turmeric rhizomes (*Curcuma longa* Linn). The potential antimicrobial and antioxidant capacity of curcumin make it a favorable option for application in active food packaging [16,17]. Additionally, some studies on animals and humans have proven its safety and nontoxicity [18]. 

The current work aimed to prepare a biodegradable active film from GE and SFTG containing curcumin using complex coacervation. The effects of curcumin on mechanical, antimicrobial, antioxidant, and barrier properties of GE/SFTG films were investigated. Moreover, curcumin release rate from the blended films into fatty food simulants (50% ethanol, *v*/*v*, that was recommended by the US Food and Drug Administration) was evaluated. 

## 2. Materials and Methods

### 2.1. Materials

Curcumin was bought from Merck (Darmstadt, Germany). TG was purchased from a local market in Isfahan, Iran. Type B GE (225 g bloom) was received from Amstel company, Netherlands. 2,2-Diphenyl-1-picrylhydrazyl (DPPH) was provided from Sigma Aldrich (Saint Louis, USA). Muller-Hinton Agar (MHA) and Muller-Hinton Broth (MHB) were purchased from QUELAB, Canada. The cultures of *Staphylococcus aureus* (ATCC:25923), *Bacillus cereus* (ATCC 11778), *Salmonella* Typhi (ATCC 19430), and *Escherichia coli* (ATCC 35150) were provided by the Pasteur Institute (Tehran, Iran). All other chemicals were of analytical grade. 

### 2.2. Preparation of Soluble Fraction of TG

A specific amount of TG (0.25% *w*/*v*) was dissolved in distilled water and kept in the refrigerator overnight to complete hydration. For separating soluble and insoluble fractions of TG, the solution was centrifuged (MPW Med, MPW-206R, Warsaw, Poland) at 5500 rpm for 1 h. Then, the supernatant was dried at 50 °C to obtain the soluble fraction of TG (SFTG) [19].

### 2.3. Preparation of Complex Coacervates

Complex coacervates of GE and SFTG were prepared according to Devi and Maji [20]. The solutions of GE and SFTG were prepared in distilled water at 50 °C, separately. In the next step, different ratios of GE (1% *w*/*w*) and SFTG (1% *w*/*w*) solutions were mixed at 50 °C (Table 1). Solutions were stirred for 30 min, followed by adjusting the pH to 3.6 using acetic acid (10% *v*/*v*). Mixtures were stirred for 1 h and kept in the refrigerator overnight. To separate the sedimented part (complex coacervates) of the mixtures, samples were centrifuged at 5500 rpm for 30 min. Eventually, the obtained complex coacervates were dried using a freeze dryer (Dena Vacuum, Tehran, Iran) at −40 °C for 24 h.

To obtain the optimum biopolymers’ ratio for the production of films, the particle yield (PY) was evaluated by the gravimetric method. To this end, the primary weight of biopolymers (M_0_) and the obtained weight of dried complex coacervates (M_1_) were measured; PY was calculated by Equation (1).

PY (%) = (M_1_/M_0_) × 100 (1)

The samples with higher PY were used for the production of films containing curcumin. 

### 2.4. Film Formation Process

The preparation of films was by casting method according to Eghbal et al., (2016) [14]. Complex coacervates powder (6% *w*/*w*) was completely dissolved in acetate buffer (pH = 3.6) at room temperature. Curcumin (0.3% dry weight) was added to the mixture and stirred overnight. Then, glycerol (35% dry weight) was added to the mixture as a plasticizer and stirred for 1 h. CaCl_2_ (1% *w*/*v*) as a cross-linking agent was also added and mixed for 5 h. The film without curcumin was prepared as a blank sample the same as above. Finally, 20 mL of the final solution was poured into a plate (90 mm diameter) and dried at room temperature (25 °C) and ambient relative humidity (RH = 45%) for 48 h. 

### 2.5. Characterization of Films

#### 2.5.1. The Thickness and Mechanical Properties

To report the mean value of thickness, five random positions of produced films were quantified using a digital micrometer (Mitutoyo, Kawasaki, Kanagawa, Japan) with the accuracy of 0.001 mm. A texture analyzer (SANTAM STM-1, Tehran, Iran) with a 50 N load cell based on the ASTM method [21] was applied to assess mechanical properties. For this purpose, strips of films in dimensions of 70 mm × 10 mm were provided. Measurements were performed with an initial gap of 30 mm and a cross-head speed of 5 mm/s. Tensile strength (TS) and elongation at break (EAB) of the samples were calculated according to Equations (2) and (3), respectively.

(2) $$\mathrm{TS}\;(\mathrm{MPa})=\frac{F}{d\times w}$$

(3) $$\mathrm{EAB}\;(\%)=\frac{E-E0}{E0}\times 100\%$$

where F is the maximum tension (N) when the films are broken, d is the thickness (mm) of films, w is the width (mm) of films, E is the length (mm) of film at rupture, and E0 is the initial length (mm) of films. The mechanical measurements were performed in triplicate, and the mean values were reported.

#### 2.5.2. Water Vapor Permeability (WVP)

The ASTM E96 method [22], based on the gravimetric method, with slight modification, was considered to determine WVP. Each test tube, with 10 mm internal diameter and 55 mm depth, contained 1 g CaCl_2_ (0% relative humidity, RH) and was sealed with the films. Then, all test tubes were placed in the desiccator with a RH = 75% and were incubated at 45 °C. To maintain 75% RH, a constant mass of a saturated solution of NaCl was used in a desiccator (Boeckel, Hamburg, Germany). The test tubes were weighed every 24 h for 7 days. Equations (4) and (5) were used to calculate water vapor transmission rate (WVTR, g/day m^2^) and WVP (g mm/kP day m^2^) of the films, respectively [23].

WVTR = Δm/(t × A)(4)

WVP = (WVTR × l)/(ΔP × (RH_1_ − RH_2_)) (5)

where l is the average thickness of the films (mm), A is the area of the test tube mouth (m^2^), ΔP is the vapor pressure difference (kPa), RH_1_ − RH_2_ is the RH difference across the films, and Δm/t is the slope of the weight change versus time (g day^−1^), which was calculated using linear regression (r^2^ ≥ 0.99).

#### 2.5.3. Moisture Content (MC) and Water Solubility

The MC was estimated gravimetrically. Each film sample (2 × 2 cm^2^) was dried in the oven at 105 °C until a stable weight. Equation (6) was applied to determine MC. The dried film samples, which were utilized to assess MC, were immersed in beakers containing 20 mL distilled water for 24 h at room temperature. The undissolved films were taken out from the water and dried at 105 °C to reach a steady weight. Equation (7) was used to compute water solubility [24]:

MC (%) = ((W_0_ − W_1_)/W_0_) × 100 (6)

Water solubility (%) = ((W_1_ − W_2_)/W_1_) × 100 (7)

where W_0_ is initial weight of film samples, W_1_ is initial dry weight, and W_2_ is final dry weight.

#### 2.5.4. The Swelling Test

To measure the swelling rate of films in water, 2 × 2 cm^2^ pieces of each sample film were separated, weighed, and soaked in distilled water. Then, the film pieces were removed from distilled water and dried with a cloth to remove excess water. The swelling degree (Equation (8)) of films was determined for 2 h with 10 min intervals by the gravimetric method [25]:

Swelling ratio (g/g) = (W2 − W1)/W1(8)

#### 2.5.5. Curcumin Release from Films

Curcumin release was evaluated in 50% ethanol as a simulant for fatty or oily foods [26]. At first, 2 × 2 cm^2^ cuts of films were placed in a beaker containing 25 mL of 50% ethanol. Sampling was performed at room temperature (25 °C) with 1 h intervals. The solution (3 mL) was pulled out and replaced by fresh 50% ethanol. The sampling continued for the next 24 h [27]. The released curcumin from the film samples was assessed at 425 nm using a spectrophotometer (JASCO, Tokyo, Japan) [28].

#### 2.5.6. Thermal Analysis

The thermal analysis of film samples and pure ingredients was conducted using a thermogravimetric analyzer (TGA-LABSYS EVO, SETARAM, Francelab, Budapest, Hungary). The experiment was performed under a nitrogen atmosphere with a 10 °C/min heating rate.

#### 2.5.7. X-ray Diffraction (XRD)

The physical state of the film samples and pure ingredients was studied by XRD pattern obtained by an X-ray diffractometer (XRD-ASENWARE, AW-XDm300, Shenzhen, China). The equipment was operated with Cu–Ka (λ = 1.54 Å) under the voltage of 40 kV and 30 mA. The diffraction angular range for all samples was 2θ = 5–60° with a step size of 2θ = 0.05°.

#### 2.5.8. Fourier Transform Infrared (FTIR) Spectroscopy

FTIR spectra of the pure ingredients and the film samples were characterized by an FTIR spectrometer (FTIR-Jasco, Tokyo, Japan). All FTIR spectra were carried out at a resolution of 4 cm^−1^ and the spectral range of 4000 to 400 cm^−1^. 

### 2.6. Antioxidant Activity of Films

The antioxidant activity of film samples with and without curcumin was estimated based on hydrogen-donating ability, using the DPPH free-radical scavenging [29]. Briefly, 2 mL of each film solution was mixed with an equal volume of DPPH ethanolic solution (50 mg/L) and incubated in the dark for 30 min. DPPH solution and ethanol were mixed and used as blank. The absorbance was recorded at 517 nm using a spectrophotometer. DPPH radical scavenging capacity was determined using Equation (9):

Free radical scavenging activity (%) = ((Abs_DPPH_ − Abs_sample_)/Abs DPPH) × 100 (9)

where Abs_sample_ and Abs_DPPH_ are the absorbances of DPPH in film solution and blank, respectively.

### 2.7. Antibacterial Activity of Films

The antibacterial activity of the film samples was tested against 2 Gram-positive (*S. aureus* and *B. cereus*) and 2 Gram-negative (*S. Typhi* and *E. coli*) of the most common food-borne pathogenic bacteria [30]. First, all strains were incubated in TSB for 24 h at 37 °C. Then, the bacterial strains were diluted in MHB to obtain McFarland turbidity standard No. 0.5 (10^8^ CFU/mL). These cultures were adjusted to the final concentration of bacterial cell suspension of 10^5^ CFU/mL by diluting in MHB. 

The antibacterial analysis was performed by agar disc diffusion and agar well diffusion. For the agar disc diffusion method, approximately 100 µL of prepared bacterial suspension was spread on solidified MHA surface by swab. The film samples were cut into a 6 mm diameter disc and placed on agar plates [31]. In addition, to evaluate antibacterial activity by agar well diffusion method, 100 µL of film solutions were poured into 6 mm diameter wells, which were punched on the plates containing MHA [32]. After 24 h incubation (37 °C), the diameter of inhibition zone was determined by a digital micrometer (Mitutoyo, Kawasaki, Kanagawa, Japan). Triplicate measurements of antimicrobial activity were performed under the same conditions for each film sample.

### 2.8. Statistical Analysis

All investigations were performed in triplicate. The mean ± standard deviation with the significant difference (*p* < 0.05) was determined by Duncan’s multiple range test (SPSS statistical software, version 20). 

## 3. Results and Discussion

### 3.1. Yield of Complex Coacervates

The PY of samples with different ratios of GE and SFTG were calculated (Table 1). The results showed a significant difference between the PY of samples (*p* < 0.05). It was varied in a range from 14.33 ± 0.81 to 60.06 ± 0.14%. As far as GE content was enhanced, PY was improved. The yield in 1GE: 2SFTG ratio was significantly lower than two other samples. According to previous studies, the ratio of polymers in the complex coacervates affects complex yield. Furthermore, the lower balance of net charge and the weak interaction leads to formation of lower complex coacervates [33,34]. Based on the acquired results, the samples with higher PY (1GE: 1SFTG and 2GE: 1SFTG) were selected to produce films.

### 3.2. Physicochemical and Mechanical Properties of the Produced Active Films

#### 3.2.1. Thickness and Mechanical Properties

The mechanical properties and thickness of all produced films are demonstrated in Table 2. There was no significant difference between the thickness of samples (*p* < 0.05). This might be related to the amount of curcumin, which was not enough to impact thickness. The authors of [35,36] also reported the same results. Mechanical properties are the key parameters in food packaging to have enough strength and flexibility to keep and carry the food [37]. As given in Table 2, there was no significant difference between film samples with curcumin (C1 and C2) and without it (F1 and F2) in TS and EAB (*p* < 0.05). After the addition of curcumin, the TS of F1 and F2 samples slightly decreased, possibly due to the intramolecular interaction of polymers with curcumin [35]. Other studies also indicated that TS declined after adding bioactive compounds [38,39,40]. By reducing TS, EAB (flexibility) increased due to the destruction of the intermolecular interaction between GE and SFTG when adding curcumin. The same finding was observed in tara gum/polyvinyl alcohol films incorporated with curcumin [41] and methylcellulose films containing α-tocopherol [38]. 

#### 3.2.2. Water Vapor Permeability

The WVP is an important parameter representing the passage of water molecules through the films [42]. The WVP of film samples (Table 2) slightly decreased after adding curcumin, but the difference was not significant (*p* > 0.05). According to previous studies, adding hydrophobic compounds into composite films led to a lower WVP. In addition, a low amount of curcumin might have less impact on WVP values [35,43,44]. 

#### 3.2.3. Moisture Content and Water Solubility

MC and WS of the film samples (Table 2) were influenced by different ratios of GE and SFTG with statistically significant differences (*p* < 0.05). The ranges of MC and WS were 42.62 ± 0.057 to 63.67 ± 0.014% and 35.93 ± 3.55. to 96.73 ± 1.11%, respectively. The samples containing more GE (F2 and C2) showed lower MC and WS than F1 and C1 samples. Additionally, a higher amount of gum in F1 and C1 samples caused an increase in MC and WS. The authors of [9] found that MC rose due to the increase in the amount of TG in films. In addition, the authors of [14] realized that adding more content of hydrophilic proteins to blended films reduced MC. Other researchers also proved that higher GE lessened WS of the film samples [45]. The authors of [46] studied chitosan/polyvinyl alcohol/fish gelatin films and demonstrated that the solubility of films reduced after the addition of fish gelatin. This was the result of reaction of polymers with hydrogen bonds, which led to lower free hydroxyl groups and lower bonding to water molecules. The control samples (F1 and F2) had significantly higher MC and WS compared to those with curcumin (C1 and C2) (*p* < 0.05). This finding was attributed to the hydrophobicity of curcumin, which influenced the MC and WS of films. Similarly, other studies also elucidated that the addition of hydrophobic compounds tends to reduce MC [36,43] and WS [35,43] of blended films. 

#### 3.2.4. The Swelling Results of the Films

**Figure 1:**
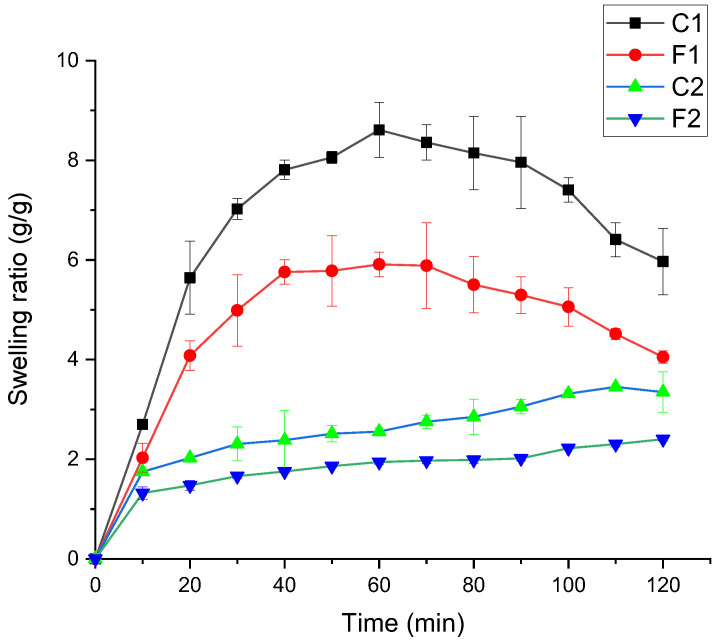
Swelling ratio of curcumin-loaded active film samples. **F1** (1GE: 1SFTG); **F2** (2GE: 1SFTG); **C1** (1GE: 1 SFTG/curcumin); **C2** (2GE: 1SFTG/curcumin).

The swelling ratio (SR) of film samples is shown in Figure 1. The proportion of GE and SFTG showed a significant effect on SR. The maximum SR was observed for the film samples with an equal ratio of GE and SFTG (F1 and C1). The samples with the highest amount of GE (C2 and F2) presented the lowest SR. The control samples (F1 and F2) were able to uptake more water than samples with curcumin because of the hydrophilicity of polymers. Lee et al., (2004) evaluated different ratios of gellan and GE composite films and concluded that SR declined at higher content of GE [45], in agreement with the SR of GE/chitosan films [47]. After the addition of curcumin, which is a hydrophobic compound, the swelling capacity was enhanced. The presence of available hydrophilic groups and crystallinity of used materials impacted swelling behavior of the film samples. A similar trend was obtained for SR of curcumin/guar gum/polyhydroxyalkanoates composite films [48]. Similarly, incorporating curcumin into different carbohydrate films exhibited a bigger SR [35]. Other researchers also reported that the water absorbance of TG and GE films mounted as the content of gum was elevated. This is the result of a greater capacity to bond with the water molecules [9]. 

#### 3.2.5. Thermal Properties

**Figure 2:**
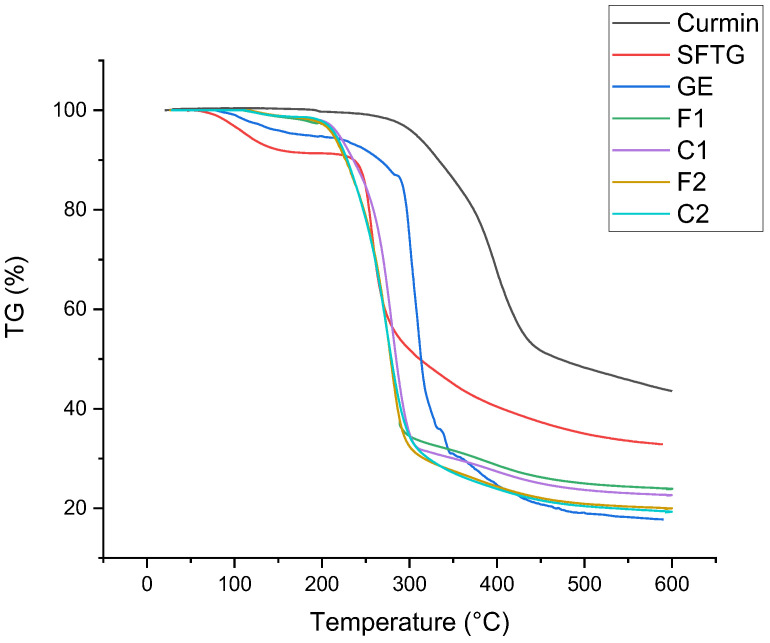
TGA of curcumin, SFTG, GE, and film samples. **F1** (1GE: 1SFTG); **F2** (2GE: 1SFTG); **C1** (1GE: 1 SFTG/curcumin); **C2** (2GE: 1SFTG/curcumin).

The thermal stabilities of pure materials and different film samples are demonstrated in Figure 2. The first weight loss that occurred at <140 °C was related to moisture evaporation. For pure curcumin, the thermal degradation started at about 270 °C and reached 470 °C. The main thermal degradation of GE took place in the range of 260 to 450 °C. The original weight loss of SFTG started at about 220 °C, and 60% weight loss happened up to 450 °C. All film samples showed similar weight loss trends. The main weight loss of films (70%) started at about 200–300 °C, which was related to the depolymerization of polymers, and the final step of thermal decomposition (10%) was owing to the decomposition of film samples [49]. Incorporation of curcumin into films did not make a considerable difference in thermal stability compared to the pure film samples. Overall, the films showed lower thermal stability than pure materials. In agreement with this outcome, other researchers did not find any remarkable difference in the thermal stability of films with or without curcumin [35,50,51].

#### 3.2.6. XRD Results

**Figure 3:**
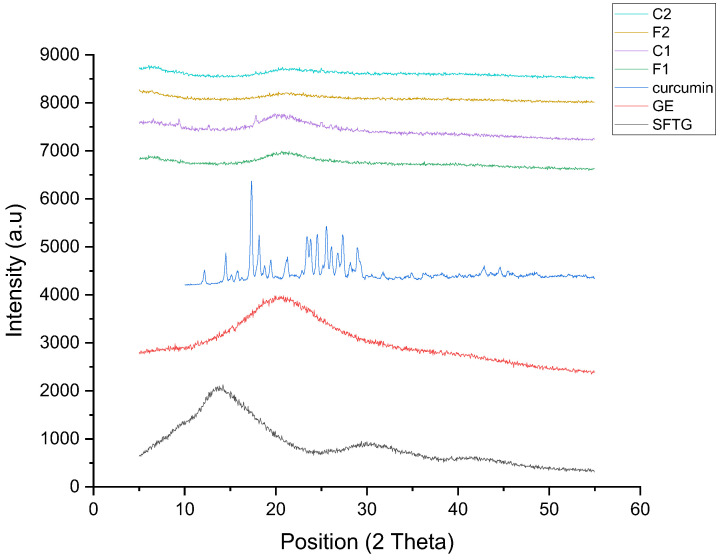
XRD patterns of curcumin, SFTG, GE, and film samples. **F1** (1GE: 1SFTG); **F2** (2GE: 1SFTG); **C1** (1GE: 1 SFTG/curcumin); **C2** (2GE: 1SFTG/curcumin).

The XRD patterns of pure materials and various film samples are shown in Figure 3 as an indicator for evaluating their crystallinity. The XRD pattern of SFTG revealed maximum peak intensity at 13.7° and a broader peak at 30.35°. This result showed the amorphous nature of SFTG with an almost crystalline (or a crystalline-like) part. A broad curve at around 2θ ≈ 20° was observed in the XRD pattern of GE. According to previous studies, this is attributed to the α-helix and triple-helical structure of GE, which causes a semicrystalline structure [52]. Regarding curcumin, some highlighted peaks appeared in the region of 2θ ≈ 12.25°–29° in its XRD pattern. Thus, the structure of curcumin was considered crystalline [53]. All film samples depicted amorphous structure, and the semicrystalline pattern of their individual components was eliminated. In addition, it was observed that the crystalline peaks of curcumin did not emerge in C1 and C2 samples. This indicated successful interaction between biopolymers and curcumin. 

#### 3.2.7. Chemical and Functional Groups of the Films

**Figure 4:**
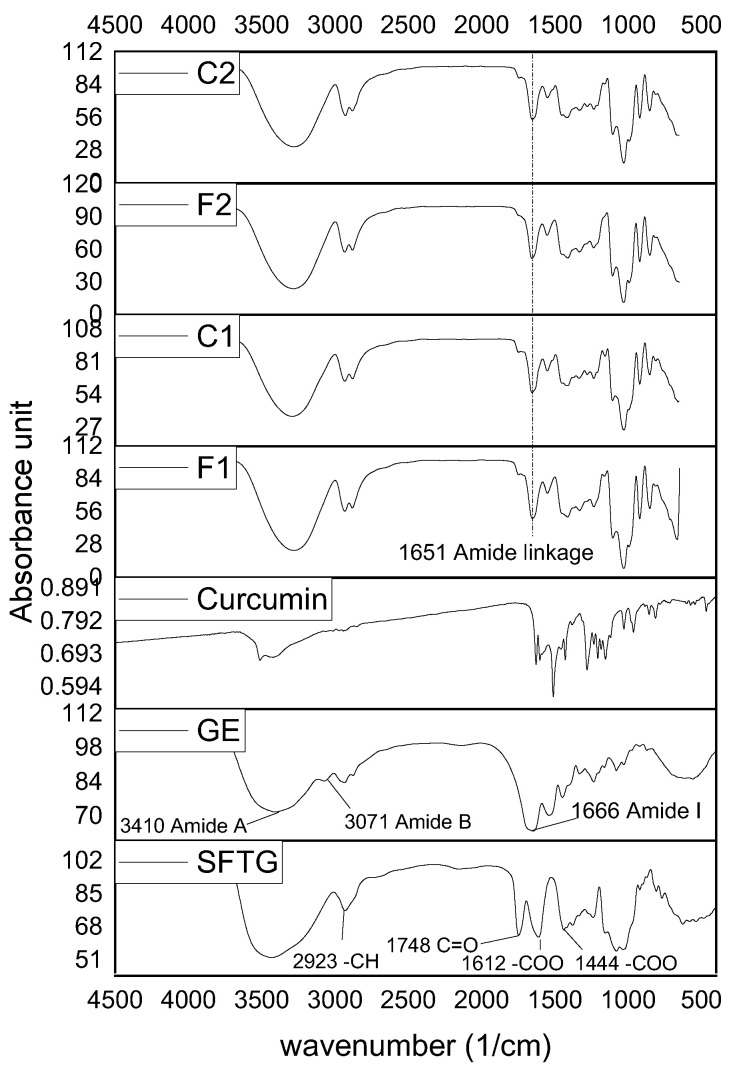
FTIR spectra of curcumin, SFTG, GE, and film samples. **F1** (1GE: 1SFTG); **F2** (2GE: 1SFTG); **C1** (1GE: 1 SFTG/curcumin); **C2** (2GE: 1SFTG/curcumin).

The functional groups of GE, SFTG, and curcumin and their interactions in the film samples were characterized by FTIR (Figure 4). In the case of GE, the peaks at around 1666, 1513, and 1232 cm^−1^ were associated with amide I (C=O and C–N), amide II (N–H and C–N), and amide III (N–H and C–N), respectively. Additionally, the absorption peaks at almost 3410 and 3071 cm^−1^ were ascribed to the amide A, and amide B of GE, respectively [20,54,55]. FTIR spectroscopy of SFTG revealed the carbohydrate characteristic peaks in the area of 908–1248 cm^−1^. The bands at about 1612 and 1444 cm^−1^ belonged to the carboxylic group (–COO). Moreover, SFTG displayed a peak at 1748 cm^−1^ which is due to the C=O group. The peaks that appeared at around 3438 and 2923 cm^−1^ corresponded to the hydroxyl (–OH) and methylene group absorption bands, respectively [56]. The curcumin spectrum depicted a band at approximately 1627 cm^−1^ that contributed to the overlapping alkenes and carbonyl groups. The peak at 1600 cm^−1^ appeared due to benzene ring stretching vibration. Furthermore, the bending vibration of the C–H group and C–O and C–O–C stretching vibration were revealed at 1431, 1280, and 1026 cm^−1^, respectively. The emerging peaks at 3419–3508 cm^−1^ were related to O–H groups [57,58]. 

All film samples showed approximately similar FTIR spectra. At first glance, it is obvious that some peaks disappeared and shifted, which might be a consequence of some chemical reactions. Accordingly, the reaction between the carboxylic group of SFTG and amide I of GE formed an amide linkage which showed a slight shift from 1666 cm^−1^ in GE to 1652 cm^−1^ in all films, respectively [59,60]. Moreover, the amide A band (3410 cm^−1^) in GE shifted in all film samples. This might be a result of bonding the hydroxyl group of SFTG and glycerol with amine groups of GE. A similar observation was found by [59,61]. Amide B did not appear in any of the film samples, which may be due to hydroxyl group interactions [59]. 

### 3.3. Release Results of Curcumin from Active Films

**Figure 5:**
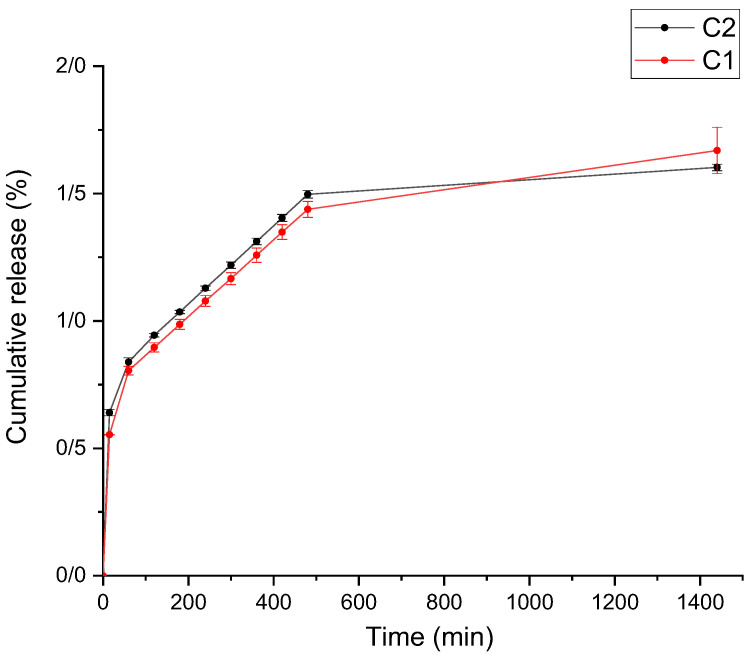
Release of curcumin from composite films in 50% ethanol fatty food simulant. **C1** (1GE: 1 SFTG/curcumin); **C2** (2GE: 1SFTG/curcumin).

The release of curcumin from the films into 50% ethanol (fatty food simulants) is elucidated in Figure 5. As is clear, the cumulative amount of released curcumin was very low and <2% of its release was after 24 h. Most of the curcumin release happened in the first 15 min. The release trend of C1 samples was very similar to C2. Various factors impact the release of bioactive ingredients from the base matrix, such as the type of polymers, the interaction of film compounds, the swelling rate, and the solubility of film samples [62]. In addition, the increasing rate of release is expected to continue after 24 h.

### 3.4. Antioxidant Activity of Curcumin-Loaded Films

**Figure 6:**
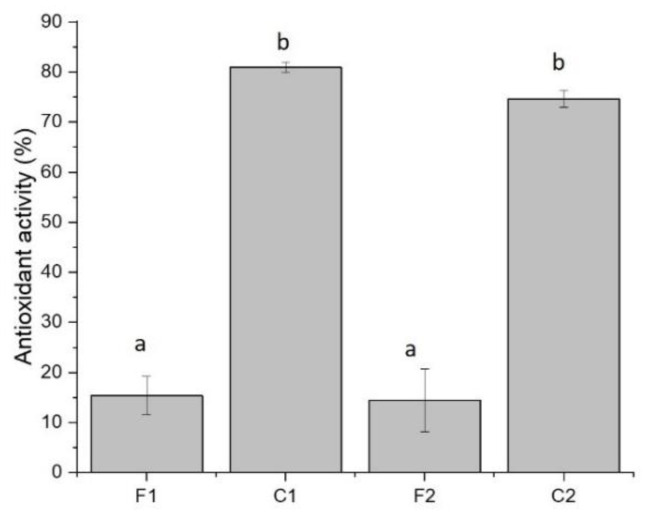
Antioxidant activity of film samples containing curcumin. **F1** (1GE: 1SFTG); **F2** (2GE: 1SFTG); **C1** (1GE: 1 SFTG/curcumin); **C2** (2GE: 1SFTG/curcumin). Different letters show significantly different (*p* < 0.05).

The pure film samples (F1 and F2) revealed approximately 15% antioxidant activity (Figure 6). After loading of curcumin, the antioxidant capacity of films improved strikingly. The antioxidant feature of C1 and C2 samples was 80.9% and 74.6%, respectively. The antioxidant capacity of curcumin corresponds to its donation of H atom from phenolic groups [63]. The proportion of biopolymers in film complexes did not significantly affect this property. Previously, it was reported that adding curcumin into carbohydrate-based films caused a noticeable rise in their antioxidant activity [35]. Moreover, a remarkable increase in antioxidant activity was found in GE/curcumin edible packaging [43,51]. Other researchers also prepared films with different biopolymers incorporating curcumin, which resulted in high antioxidant activity [41,64,65]. 

### 3.5. Antibacterial Activity of Curcumin-Loaded Films

The film samples showed no antibacterial activity against tested bacteria. This result could be related to the low concentration of curcumin in the studied films. Moreover, the interaction of curcumin with biopolymers may hinder the release of curcumin, which results in a lack of antibacterial activity. Musso et al., (2016), who surveyed the smart GE edible films containing 0.02 *w*/*v* curcumin, detected similar findings [43]. In another study, researchers indicated that sunflower protein films incorporated with phenolic compounds of sunflower seeds did not affect bacterial strains because phenolic compounds were bonded with protein [66]. The antibacterial effect of curcumin that incorporated poly (butylene adipate-co-terephthalate) was also insignificant, although it revealed a slight enhancement by raising the amount of curcumin [65]. In other studies in which curcumin content was higher than this research, a considerable antimicrobial consequence was observed against Gram-positive and Gram-negative bacteria [40,50]. 

## 4. Conclusions

Active packaging films were made by complex coacervation of SFTG and GE loaded with curcumin. Although the curcumin-loaded films did not show any antibacterial activity, they presented a great antioxidant activity (around 80%). This result could be attributed to the low concentration of curcumin in the studied films, and higher concentrations of curcumin in active packaging are suggested to improve the antimicrobial activity. The cumulative release of curcumin from films in 50% ethanol fatty simulants increased steadily during 24 h. The present work outcomes revealed the potential use of the obtained film samples in antioxidant packaging to prolong the shelf-life of fatty food due to the high efficiency of the released curcumin.

## Figures and Tables

**Table 1:** Different formulations of complex coacervates between gelatin (GE) and soluble faction of tragacanth gum (SFTG).

Biopolymer Ratios (*w*/*w*)	Particle Yield (%)
1GE: 1SFTG	46.57 ± 1.71 ^b^
2GE: 1SFTG	60.06 ± 0.14 ^a^
1GE: 2SFTG	14.33 ± 0.81 ^c^

Different letters indicate significant differences (*p* < 0.05).

**Table 2:** Physical and mechanical properties of curcumin-loaded active film samples.

Film Samples	Thickness (mm)	WVP (g mm/kP Day m^2^)	Moisture Content (%)	Water Solubility (%)	Tensile Strength (MPa)	Elongation at Break (%)
F1 (1GE: 1SFTG)	0.21 ± 0.06 ^a^	0.6048 ± 0.12 ^a^	63.67 ± 0.014^d^	96.73 ± 1.11^d^	1.74 ± 0.21 ^b^	81.48 ± 2.91 ^a^
C1 (1GE: 1SFTG/curcumin)	0.21 ± 0.02 ^a^	0.4752 ± 0.15 ^a^	54.14 ± 0.026 ^c^	80.74 ± 1.21 ^c^	0.62 ± 0.06 ^a^	122.00 ± 11.81 ^b^
F2 (2GE: 1SFTG)	0.25 ± 0.04 ^a^	0.6912 ± 0.14 ^a^	50.22 ± 0.025 ^b^	78.80 ± 8.60 ^b^	1.77 ± 0.48 ^b^	98.87 ± 23.08 ^a^
C2 (2GE: 1SFTG/curcumin)	0.25 ± 0.04 ^a^	0.6048 ± 0.12 ^a^	42.62 ± 0.057 ^a^	35.93 ± 3.55 ^a^	0.17 ± 0.07 ^a^	109.58 ± 10.78 ^b^

Different letters indicate significant differences (*p* < 0.05).

## Data Availability

Data is contained within the article.
